# Recovering the Good Earth: China’s Growing Organic Market

**DOI:** 10.1289/ehp.116-a346

**Published:** 2008-08

**Authors:** David A. Taylor

People whose impressions of Chinese food exports are colored by reports of contaminated pet food or pesticide-laced dumplings might not expect China to have much of an organic market. They would be wrong. According to “The Greening of China’s Food: Green Food, Organic Food, and Eco-labelling,” a paper presented at the May 2008 Sustainable Consumption and Alternative Agri-Food Systems Conference, 28% of China’s arable land—just over 34 million hectares—is devoted to “eco-foods,” a designation that includes organic certification as well as China’s unique “green” and “hazard-free” categories of food.

In an increasingly global food market, unexpected opportunities for farmers around the world are opening in unlikely places, such as China’s experiments with organic food. The world market for organic foods has soared, with sales growing by more than US$5 billion annually and doubling between 2000 and 2006, according to the International Federation of Organic Agriculture Movements (IFOAM), an umbrella organization based in Bonn, Germany. That international trend explains why China and other countries—despite relatively little domestic demand for organic food—have become big players in the organic market.

Organic farming in China is largely an export-oriented industry, with the main products for both export and import being cereals, soybeans, and tea, followed by some vegetables, fruits and meat. Yet, although China has instituted several eco-food certification systems, reports of lax inspections persist. “Regulation is an imperfect substitute for the accountability, and trust, built into a market in which food producers meet the gaze of eaters and vice versa,” author Michael Pollan writes in his 2008 book *In Defense of Food: An Eater’s Manifesto*. In situations where farmers and consumers can’t meet face-to-face at the farmer’s market, however, some form of inspection is all that’s left to serve the interest of public health. How, then, is China adapting to secure the safety of its exports and the health of its own citizens?

## Beating a Bad Rap

Many observers, including Pollan, are skeptical of China’s organic exports. “You cannot count on the regulatory regime in China,” he said on National Public Radio’s *Talk of the Nation* on 10 August 2007. For Pollan, China’s poor record on environmental protection raises a critical question: just how “organic” in actuality are China’s organic exports? Expanding on this concern in *In Defense of Food*, he writes that in China “the rapid industrialization of the food system is leading to alarming breakdowns in food safety and integrity.”

Such concerns are reinforced by incidents such as the January 2008 discovery that meat dumplings exported to Japan from Hebei Province were contaminated with methamidophos, an organophosphate pesticide that can be highly toxic if ingested orally. The dumplings caused nausea and dizziness in 175 people, and Japanese supermarkets temporarily removed all Chinese meat products from their shelves. (Chinese authorities have suggested the methamidophos was applied in Japan as product sabotage, contradicting Japanese police findings.)

Many U.S. consumers wonder whether China’s soil and air are too polluted to support truly organic agriculture. Greg Fogel, a master’s degree researcher in public policy and natural resources at the University of Michigan in Ann Arbor, says “it depends on the company” as to whether organic certification accounts for nonagricultural pollutants in the soil. More broadly, Fogel and others say that China’s organic farming industry is focused on remote provinces where urban industry emissions are relatively scarce and where “modern” agrochemicals are not available. However, in the more urban and industrial south near Hong Kong, up the Guangdong coast and in the Yangtze Valley, soil pollution is a recognized problem.

Chinese food production has a bad rap within the country as well. The Chinese government estimates that food poisoning affects up to 40,000 people each year, according to *The Spread of Organic Agriculture in China*, a research brief by Natalie Baer published in November 2007 by the nonprofit Woodrow Wilson International Center for Scholars.

Health has become a vital public issue in China, and protecting children becomes especially important in a land with a one-child policy, notes Brian Heimberg, an employee of a regional planning and design agency in Shanghai who previously worked for several years as business development officer for wholesaler Shanghai Organics. He says consumer suspicion of central labeling and claims is widespread, and incident reports get amplified by word of mouth. Yet Fogel, during the nine months in 2007 he spent as community outreach and education coordinator for Shanghai Organics, found that most of the company’s customers viewed “organic” as a foreign idea not necessarily linked to their own concerns about food safety. “The two biggest barriers [to market growth in China] still are trust and price,” he says.

A second obstacle to domestic acceptance of organic food remains the difference in price compared with conventional produce. In 2004, organic foods in the United States commanded premiums of 9–78% over comparable nonorganic items, according to John Stevens-Garmon and colleagues in volume 22, issue 2 (2007) of *Choices*, a food trade magazine. In China—where the eco-food market started in cities and expatriate communities that enjoy incomes much higher than local wages—that premium can be up to 700% over comparable items, according to IFOAM.

Still, organic retailers have begun to multiply in China in the past year, and according to Ursula Chen, formerly a consultant to the U.S. Agricultural Trade Office in Guangzhou, nearly all supermarkets in mainland China have doubled their floor space for organic goods. Imported organic products are also available in some high-end retail stores. “A conclusion that can be made is that more local Chinese are buying organic,” she says.

## Toward an Optimal Policy

For several years China has expressed commitment to improving its environmental record and that of its agricultural sector, but it is still exploring how to make good on this promise. “At the national level, a lot of it is rhetoric,” says Baer. In China’s policy discussion, she says, Beijing has taken its cues from the global market with an eye to export potential.

Domestically, China’s policy experiment involves testing the international organic approach against its own categories of green and hazard-free food, which the government introduced in the early 1990s and 2001, respectively, in response to concerns over health incidents and contaminated food. Green certification indicates that chemicals used on the produce have been strictly controlled and the food is guaranteed to be safe to eat (initially the term was “pollution-free” but that seemed to imply that normal food contained pollution, so the name changed). Hazard-free certification focuses on controlling illegal use of highly toxic agricultural chemicals and violations of pesticide residue standards.

As the promise of the global organic market emerged, the State Environmental Protection Administration (SEPA) established an agency to set standards for exports modeled on international standards. In 1994 this agency became the Organic Food Development Center (OFDC), based in Nanjing. Organic exports certified with the OFDC label have grown since China joined the World Trade Organization in 2001. That year the OFDC instituted the first comprehensive standard for inspecting and certifying organic products, according to center staff scientist Xie Weihua and colleagues in a paper presented at the December 2007 Regional Conference on Organic Agriculture in Asia.

In 2002 China signaled the rising priority of the organic products sector by creating another agency, the Certification and Accreditation Administration of China (CNCA), to take over the growing new field of accreditation nationwide. By early 2005, that agency had established the Chinese National Organic Product Standard (CNOPS) under a separate law for organic products to resolve confusion over the competing eco-food standards. CNOPS is based on the IFOAM Basic Standards—a set of principles, recommendations, and baselines to ensure organic integrity—and is compatible with U.S., European, and Japanese rules. That same year, according to OFDC figures, the acreage devoted to certified organic farming had increased nearly tenfold in just three years, and reached one-sixth of the area devoted to green food.

By late 2007, CNCA oversaw the inspections and licensing of close to 30 decentralized agencies that certify farms for eco-food production. OFDC remains the most commonly used certifying agency, but other certifying bodies and their labels have proliferated as organic farms choose an inspecting agency according to the export destination of their produce, according to Xie. Baer explains that what might appear to be a bureaucratic overlap between the two ministries’ efforts became part of the policy experiment. “This duality isn’t uncommon,” she explains. China may be using this parallel tracking (and inherent competition between the two systems) to define its options for an optimal policy.

## Local Variations

At the province level, governments are tinkering with supports that attract organic farming. Some provinces have created incentives for organizing their own large export ventures. Other jurisdictions offer incentives to private operators; the district government offered a subsidized rental rate to Shanghai Organics for its farm operation, says Heimberg, and its farm received other tax benefits. In exchange, the local government used the farm as a demonstration of what the district could become; Heimberg comments, “In China, as other places, relationships are of utmost importance.”

But distance to markets can limit the scope of local incentives. In Yunnan Province, for example, provincial policy differs little from national policy, according to Marco Stark, an inspector for FLO-CERT, a company that certifies Fairtrade worker and environmental practices. But even in Yunnan’s capital, a city of nearly 5.8 million people, there’s next to no market for organic food, according to Stark, and Yunnan farmers face barriers to entering distant urban markets in Beijing, Shanghai, and Hong Kong.

Another factor limiting the domestic distribution of organic produce is that so much of the supply is being exported. The stores selling organic goods—including the French-based supermarket Carrefour, among other chains—need a steady high-volume supply to maintain consumer confidence. Without a steady supply, small producers can’t enter the market the way large-volume exporters can.

In Hong Kong, it’s a different story. The island’s citizenry have a relatively high income and a health consciousness that makes them willing to pay a premium for safe food. The U.S. Department of Agriculture (USDA) has even identified Hong Kong as a potential market for U.S. organic exporters, according to *South China Organic Food Market Brief*, a 2006 report prepared by Chen, which notes that many farm businesses had flourished selling organic and green produce in supermarkets in South China. Chen says many consumers in this region are alarmed by reports that China uses 30% of the world’s nitrogen fertilizer on under 10% of the world’s arable land, and is the world’s largest user of pesticides and chemical fertilizer. In 2006, she says, there were 9 companies in Guangdong (the large mainland city near Hong Kong) selling 74 types of certified organic products—although most of the 2,213 tons sold were bound for export.

Organic awareness in Hong Kong goes beyond the produce to the soil where it grows. The Hong Kong Organic Resource Centre, which began certifying organic producers in 2004, requires soil samples from every farm applicant, according to Sharon Chan, a staff scientist at the center. The samples are tested for residual levels of heavy metals and other contaminants using criteria based on Dutch standards.

## Building Awareness

Economic observers including Nicholas Lardy, a senior fellow at the private Peterson Institute for International Economics, have cited the need for China to increase its domestic consumption of goods and services to achieve balanced economic growth. In this regard, it is reasonable to expect that growth in the green and organic markets could be economically as well as environmentally beneficial, and green food may be the gateway to popularizing the stricter organic market.

When Fogel was working for Shanghai Organics, his big challenge was linking the company to farmer’s markets around Shanghai. Once he got a few going, customers became interested. “One thing the Chinese consumer really likes is to examine their choices in great detail,” says Fogel, whether that means hefting cucumbers or inspecting fruit for bruises. The farmers’ market interface defused skepticism about the organic label as consumers welcomed healthier options.

Jane Tsao, director of public relations and events for BIOFarm (formerly the O Store), an organic retailer in greater Shanghai, agrees that consumer awareness is critical. She suggests that the government support consumer educational efforts such as Roots & Shoots, a nongovernmental organization founded by naturalist Jane Goodall to raise awareness among young people about environmental and humanitarian issues.

Recent growth has been impressive. A German public–private partnership between Nürnberg Global Fairs and the German Development and Investment Society has resulted in two large annual expositions to promote organic food in China. The second BioFach China event, held in Shanghai in May 2008, drew more than 9,100 visitors. Barbara Böck, a BioFach spokesperson in Bonn, says the CNOPS regulation is providing “the correct foundation for domestic market development,” with double-digit annual growth expected in the sector for the next few years.

Nonetheless, to convert that awareness to thriving small farms will require more innovative government support for those small producers. “The government claimed that now most farmers could form groups and produce and market their products,” says Stark, “[but] I don’t think that it has become much easier for the small-scale farmer yet.” Government support could remedy this situation by lowering the threshold for farmer groups to participate in government programs that support organic exports and by training farmers in organic techniques and marketing.

At this point, growers still have very limited knowledge of organic standards or farming techniques. “Organic agriculture appears to be largely in a stage of avoidance,” says Heimberg—farmers run their operations as usual, simply removing any chemical amendment listed as prohibited by the organic certification lists.” In Heimberg’s experience, Shanghai Organics farmers had little knowledge of how to choose appropriate seed, improve soil fertility with nitrogen-fixing legumes, or otherwise manage fertility without chemicals.

Chen’s report for the USDA confirms this picture in the south: many farmers and officials interpreted “organic agriculture” to mean farming as it was done in the past, she wrote, when “everything was done naturally.” Unfortunately, this understanding does not include newer techniques for maintaining soil fertility to sustain higher yields or for averting losses to pest infestations.

Baer believes this problem has roots in the centralized nature of China’s decision making. “A lot of these townships are run like corporations,” she says. “Farmers are told what to do instead of why.”

## International Cues

This year’s rise in global food prices, caused mainly by higher fuel costs and increased use of food crops for production of biofuels, has caused concern among international agencies and humanitarian organizations [see “Food vs. Fuel: Diversion of Crops Could Cause More Hunger,” *EHP* 116:A254–A257 (2008)]. In June 2008, the Food and Agriculture Organization of the United Nations held an international meeting in Rome to address food security and the impact of rising costs and climate change on the world’s poorest communities. UN officials called for urgent action and funding to increase the world’s food production by 50% by 2030.

The connections between the niche market of organic food, which only a small portion of consumers can afford, and the wider problem of food security emerged in the declaration from that Rome conference, signed by ministers and heads of state from 180 countries. Among the means toward world food security, the leaders called for medium-and long-term measures to increase investment in food research and to increase the resilience of food production systems by, among other things, maintaining bio-diversity, an important element of many organic farming systems. The leaders also encouraged more liberal international trade policies to give farmers in developing countries new opportunities to sell their products on world markets.

Also in June 2008, IFOAM, at its 16th Organic World Congress, reaffirmed by vote the principles upon which international standards for organic certification should be built: that organic agriculture must protect, sustain, and enhance human and ecological health, and that it must be managed in a fair and precautionary manner. IFOAM is working toward harmonization among different countries’ standards for organic certification in the face of a growing global market. The European Union, the United States, and Japan remain the markets for most organic products, so the rules in those three markets will significantly influence how international standards develop.

## Figures and Tables

**Figure:**
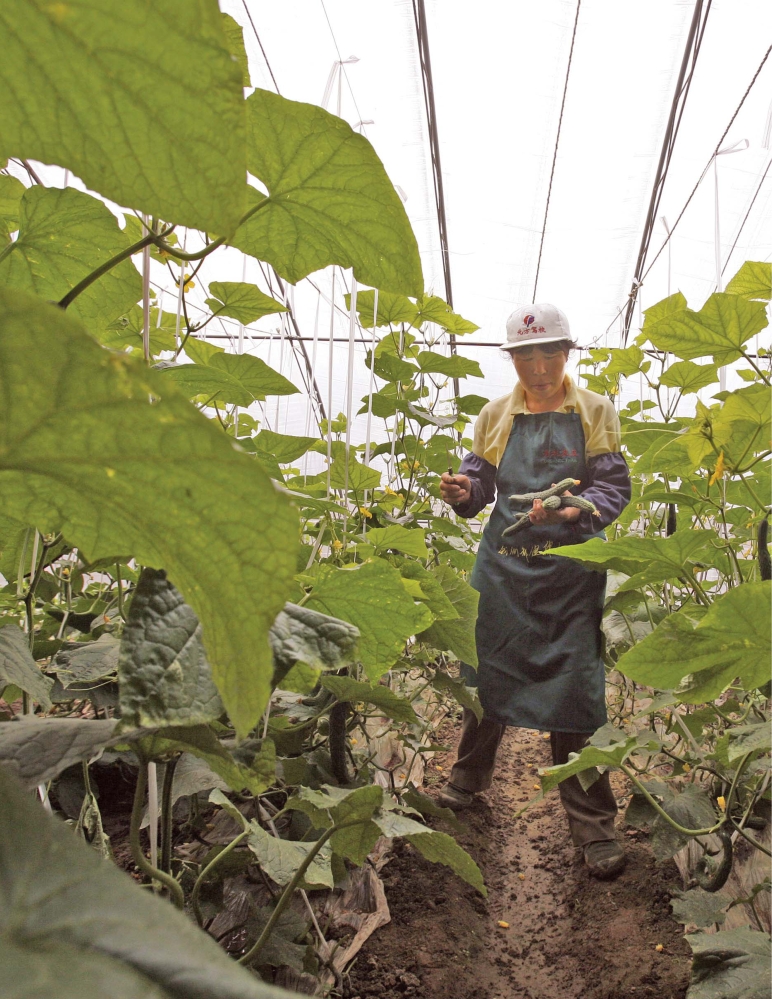
A worker picks cucumbers at an organic farm near Beijing, 20 June 2008. Typically the fruits and vegetables grown here are exported; however, during the Olympic Games in August, the farm will supply produce to event caterers.

